# Triclosan Caused Oocyte Meiotic Arrest by Modulating Oxidative Stress, Organelle Dysfunctions, Autophagy, and Apoptosis in Pigs

**DOI:** 10.3390/ani15060802

**Published:** 2025-03-12

**Authors:** Ning Zhao, Anli Xu, Jingxian Yang, Jianan Zhao, Junhao Xie, Bugao Li, Jiaxin Duan, Guoqing Cao

**Affiliations:** 1 College of Animal Science, Shanxi Agricultural University, Jinzhong 030801, China; z18434764303n@163.com (N.Z.); 13571686414@163.com (A.X.); 15129168320@163.com (J.Y.); yesterday956@163.com (J.Z.); 13028600312@163.com (J.X.); jinrenn@163.com (B.L.); 2 Shanxi Key Laboratory of Animal Genetics Resource Utilization and Breeding, Jinzhong 030801, China

**Keywords:** apoptosis, autophagy, oocyte maturation, oxidative stress, triclosan

## Abstract

Triclosan (TCS) is a highly effective broad-spectrum antibacterial agent; however, the specific roles of TCS in oocyte maturation remain poorly understood. This research investigated the influence of TCS on biologically active processes during the in vitro maturation of porcine oocytes. Our results demonstrated that TCS significantly decreased the maturation rate of porcine oocytes in a concentration-dependent manner and impaired cumulus expansion. These detrimental effects were mediated by the disruption of mitochondrial function and distribution, leading to oxidative stress characterized by an accumulation of reactive oxygen species (ROS), a decrease in the expression of the antioxidant enzymes SOD2 and GSH, reduced ATP production, and a loss of mitochondrial membrane potential (ΔΨm). We also observed interference with endoplasmic reticulum (ER) distribution, disturbances in Ca^2+^ homeostasis, and fluctuations in ER stress, as evidenced by reduced expression of ER stress-related proteins. Furthermore, TCS exposure induced autophagy, as indicated by the levels of SQSTM1 (P62) and LC3-II. Additionally, TCS increased apoptosis rates, corresponding with a downregulation of Bcl-2 expression. Collectively, our findings suggest that exposure to TCS can impair cytoplasmic function, thereby affecting oocyte quality.

## Simple Summary

Triclosan, a common antibacterial agent found in everyday products like soaps and toothpaste, has sparked concerns regarding its potential influence on reproductive health. This study investigated how triclosan exposure affects the maturation of pig egg cells, which are crucial for successful reproduction. Researchers exposed pig egg cells to different levels of triclosan during their growth in the lab and observed how it impacted their development. The results showed that triclosan reduced the ability of the egg cells to mature correctly and caused damage to key cellular structures, such as mitochondria (the energy-producing parts of the cell) and the endoplasmic reticulum (involved in protein production and calcium regulation). Additionally, triclosan increased harmful oxidative stress, triggered self-destruction mechanisms in the cells, and disrupted their ability to repair damage. These findings suggest that triclosan exposure can harm egg cell quality, potentially affecting fertility and reproductive health. This study highlights the need for further research into triclosan’s safety and its implications for human and animal health, offering critical insights for policymakers and the general public.

## 1. Introduction

Ovarian follicles promote the growth and maturation of oocytes, allowing the development of meiotic capability [1]. During the meiosis I (MI) phase, microtubules assemble into a spindle, and chromosomes align at the equatorial plate. After the first polar body (PBI) is extruded, meiotic progression halts [2]. Therefore, developing high-efficiency techniques for porcine oocyte maturation is essential for advancing female reproductive technologies. Production of porcine embryos through in vitro methods is significant for both agricultural applications and biomedical studies. In vitro maturation (IVM) is commonly employed to generate mature oocytes; however, these oocytes’ quality and developmental capabilities are often lower when grown in vitro culture (IVC) systems. This discrepancy is primarily attributed to the elevated levels of reactive oxygen species (ROS) [3] in IVM oocytes, which can lead to mitochondrial dysfunction [4] and endoplasmic reticulum (ER) stress [5], ultimately leading to oocyte apoptosis [6].

Triclosan (TCS), a chlorinated aromatic compound of synthetic origin, is extensively utilized in antibacterial products due to its potent antimicrobial activity [7]. Recent studies suggest that environmental chemical exposure, particularly to endocrine-disrupting chemicals (EDCs), is associated with reproductive health issues [8]. These chemicals may impact reproductive health by influencing hormone production, metabolism, and non-endocrine-related pathways [9].

TCS may lead to reproductive issues in both males and females [10]. In mature male Wistar rats, a low dose of TCS (40 mg/kg) already demonstrated significant toxic effects, while a high dose (320 mg/kg) led to nearly complete functional impairment of the epididymis and seminal vesicles, accompanied by a marked decline in sperm quality [11]. A series of prospective studies categorized TCS exposure into three levels based on urinary concentrations in male participants. The results indicated a notable link between the highest TCS exposure levels (>1.8 ng/mg creatinine) and a rise in infertility, showing a positive relationship between TCS exposure concentrations and infertility, while also revealing a negative relationship with fertility [12]. Additionally, a study found that TCS could adversely affect testicular development and spermatogenesis in adult zebrafish by altering sex hormone levels [13]. There are also indications that TCS exposure might impact ovarian function and ovarian reserve [14,15]. A zebrafish study demonstrated that high-dose TCS induced ovarian oxidative damage, accelerating ovarian cell apoptosis [16]. Previous research has indicated that TCS disrupts energy metabolism by altering glucose pathways, impairing steroid hormone biosynthesis and hormonal balance, and ultimately affecting female reproductive development [17]. Although the impact of TCS has been reported in female rats [18], its influence on porcine oocyte maturation remains poorly understood.

In this research, we examined the impact of TCS on porcine oocyte maturation and clarified the associated toxicological mechanisms. Our findings indicate that TCS exposure is related to oxidative stress, mitochondrial dysfunction, autophagic processes, endoplasmic reticulum stress, and early apoptosis. This research establishes a scientific basis for understanding the mechanisms that contribute to TCS-induced toxicity in oocyte quality.

## 2. Materials and Methods

### 2.1. Chemicals

All chemical reagents were obtained from Glpbio (Montclair, CA, USA), and staining kits were provided by Beyotime Biotechnology (Shanghai, China). Hormones used in the study were manufactured by Ningbo No. 2 Hormone Factory (Ningbo, China). Primary antibodies, including anti-SOD2 (1:1000), anti-GRP78 (1:3000), anti-CHOP (1:1500), anti-SQSTM1 (1:1000), and anti-LC3 (1:1000), were sourced from Proteintech (Wuhan, China), GAPDH (1:2000) was sourced from Servicebio (Wuhan, China), unless otherwise indicated.

### 2.2. Culture of the Porcine Oocytes

Ovarian samples were acquired from slaughterhouses located in Shanxi Province, China. The collected ovaries were placed in phosphate-buffered saline (PBS) buffer containing 1% penicillin and streptomycin, pre-warmed to 38 °C, and returned to the laboratory within 1–1.5 h [19]. Follicular fluid was extracted from healthy follicles (3–5 mm in diameter) using a 16-gauge needle [20]. After removing the supernatant, oocytes exhibiting an intact cumulus-oocyte complex (COC) and homogeneous cytoplasm were identified under a stereomicroscope (SMZ1270; Nikon, Tokyo, Japan) for subsequent experiments.

Every 100 COCs were transferred into 1000 µL maturation culture medium, consisting of M199 (Gibco, Waltham, MA, USA) supplemented with 0.91 mmol/L sodium pyruvate, 10 IU/mL pregnant mare serum gonadotropin, 0.57 mmol/L L-cysteine, 10 IU/mL human chorionic gonadotropin, 1 mg/mL polyvinyl alcohol, 3.05 mmol/L glucose, 10 ng/mL epidermal growth factor, 10 IU/mL follicle stimulating hormone, 10% porcine follicle fluid, 10% FBS (Gibco, Waltham, MA, USA). TCS was dissolved in DMSO to prepare stock solutions and added to the culture medium at a 1:1000 volume ratio to achieve the final concentrations (0, 2, 20, 40, and 60 μmol/L). The 0 μmol/L TCS group, serving as the control, contained an equivalent volume of DMSO (0.1% *v*/*v*) as the TCS-treated groups. The cultures were placed in Petri dishes and incubated at 38.5 °C with 5% CO_2_ for 44 h [21].

### 2.3. Assessment of Maturation in Oocytes

The assessment of cumulus expansion involved culturing groups of cumulus-oocyte complexes (COCs) for 44 h, after which the degree of expansion was evaluated and classified using a stereomicroscope (SMZ1270; Nikon, Tokyo, Japan). A subjective scoring method for assessing cumulus expansion was developed based on previously published studies. Specifically, the expansion was classified into three grades based on the relative diameter compared to the denuded oocytes: Grade A (>3× the diameter of denuded oocytes), Grade B (2–3× the diameter of denuded oocytes), and Grade C (<2× the diameter of denuded oocytes) [22,23].

Following evaluation, COCs were treated with PBS and gently pipetted 200 times to remove cumulus cells. Denuded oocytes were washed thoroughly with PBS, stained with Hoechst 33342 (Solarbio, Beijing, China), and examined under a fluorescence microscope (ECLIPSE TS 2R; Nikon, Japan). We observed that mature oocytes displayed two bright chromatin spots. Oocytes exhibiting two distinct chromatin spots, indicative of the metaphase II (MII) stage, were classified as mature.

### 2.4. Assessment of Mitochondrial and ER Distribution in Oocytes

Mitochondrial and ER distribution in oocytes were assessed using Mito-Tracker Green and ER-Tracker Red, respectively. Oocytes were treated with M199 medium containing the dyes at 38 °C in the dark for 40 min, followed by three washes with M199 to remove excess stain. Fluorescence microscopy (ECLIPSE TS 2R; Nikon, Japan) was employed to capture images. Under normal conditions, mitochondria and ER exhibited a homogeneous distribution within the cytoplasm. The percentages of oocytes displaying uniform mitochondrial and ER distribution patterns were quantified using the same methodology. At least 30 oocytes were analyzed per group, and the experiments were repeated three times.

### 2.5. Measurement of ROS, GSH, and Calcium Levels in Oocytes

The DCFH-DA probe was diluted at 1:1000 in TCM-199 to prepare the ROS working solution. Denuded oocytes were exposed to the solution and maintained at 38.5 °C under dark conditions for 30 min to facilitate probe loading. For the measurement of GSH, denuded oocytes were incubated in a medium containing 10 µmol/L Cell Tracker Blue for 40 min. The Fluo-4 AM stock solution was diluted at 1:1500 in PBS to prepare the working solution. Denuded oocytes were exposed to the solution and maintained at 38.5 °C under dark conditions for 30 min to facilitate probe loading. Fluorescence images were obtained with a fluorescence microscope (ECLIPSE TS 2R; Nikon, Japan), and fluorescence intensity was quantified using ImageJ (version 1.54f) with background subtraction. At least 30 oocytes were analyzed per group, and the experiments were repeated three times.

### 2.6. Determination of ATP Levels

ATP concentrations were measured with the ATP Assay Kit. A group of 180 denuded oocytes was placed in a centrifuge tube containing 30 µL of cell lysis buffer and underwent three cycles of freezing and thawing in liquid nitrogen to ensure complete cell disruption. The lysate was divided into 10 aliquots for analysis. Luminescence was immediately recorded using a luminometer (BioTek, Winooski, VT, USA) for 10 s, and ATP content was determined based on a standard curve generated using ATP standards provided in the kit. At least 60 oocytes were analyzed per group, and the experiments were repeated three times.

### 2.7. Mitochondrial Membrane Potential (ΔΨm) Assessment

A working solution, prepared by diluting the JC-1 reagent 200-fold, was used to incubate oocytes under dark conditions for 35 min. Following incubation, oocytes were rinsed with JC-1 buffer. Fluorescence images were captured using a fluorescence microscope (ECLIPSE TS 2R; Nikon, Japan). The proportion of red fluorescence (associated with aggregates) to green fluorescence (linked to monomers) was quantified using ImageJ software (version 1.54f) with background subtraction to determine changes in ΔΨm. At least 30 oocytes were analyzed per group, and experiments were repeated three times.

### 2.8. Assessment of Early Apoptosis in Oocytes

Annexin V-FITC was diluted at 1:15 with binding buffer, and denuded oocytes were incubated in the solution away from light for 40 min. Oocytes were washed to remove the unbound dye, and green fluorescence signals were acquired using a fluorescence imaging system. At least 30 oocytes were analyzed per group, and experiments were repeated three times.

### 2.9. Western Blotting

Sixty oocytes were dissolved in 12 µL of RIPA Lysis Buffer (Beyotime, Shanghai, China) containing 1% phenylmethylsulfonyl fluoride (PMSF). Subsequently, 5× SDS-PAGE loading buffer was added according to the specified proportion, and the mixture was boiled for 10 min. Total protein lysates were separated by SDS-PAGE and transferred onto nitrocellulose (NC) and polyvinylidene difluoride (PVDF) membranes for further analysis. The membranes were incubated with a blocking buffer (Servicebio, Wuhan, China) for 15 min. Following this, the membranes were incubated overnight at 4 °C with primary antibodies. After the membranes were treated, they were incubated for one hour at room temperature with goat anti-rabbit antibodies conjugated to HRP (dilution 1:20,000; LI-COR, Lincoln, NE, USA) while gently shaking. After washing, the protein bands were visualized on X-ray film using ECL Plus (ODYSSEY CLx, LI-COR Bioscience, Lincoln, NE, USA).

### 2.10. Statistical Analysis

All experiments were conducted with three independent biological replicates. Data analysis was performed using GraphPad Prism 9.5 (San Diego, CA, USA), and results are presented as mean ± SEM. Differences were considered statistically significant at *p* < 0.05.

## 3. Results

### 3.1. TCS Impedes Nuclear Maturation in Oocytes

**Figure 1:**
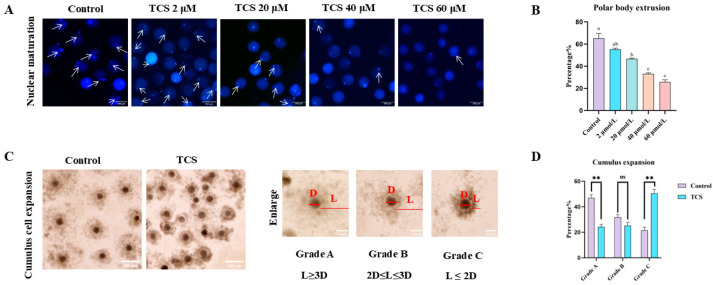
TCS impedes nuclear maturation in oocytes. (**A**) Representative micrographs of nuclear staining treated with different concentrations of TCS (2, 20, 40, 60 μmol/L). MII oocytes are indicated by white arrows. Scale bar = 100 µm. (**B**) Rate of the first polar body extrusion in different groups. Distinct letters positioned above the columns signify significant differences. (**C**) Representative morphology of COCs expansion in the control and TCS groups. “D” represents the diameter of the oocyte, and “L” represents the diffusion diameter of the cumulus cells. Scale bar = 300 µm, Enlarge: Scale bar = 100 μm. (**D**) The proportion of different grades of cumulus expansion in the control and TCS groups (ns *p* > 0.05, ** *p* < 0.01).

To examine the harmful impacts of TCS on oocytes, we assessed oocyte maturation in response to various concentrations (2, 20, 40, and 60 µM) of TCS treatment after a culture period of 44 h, which is when the majority of oocytes are expected to achieve MII. We assessed PBI extrusion as an indicator of oocyte maturation. Successful PBI extrusion occurred in the majority of control oocytes, whereas a significant number of oocytes exposed to TCS exhibited failure in this process (Figure 1A). The statistical analysis indicated that the rate of polar body extrusion was significantly reduced at TCS concentrations of 20 µmol/L, 40 µmol/L, and 60 µmol/L (20 µmol/L: 46.66 ± 0.62, *p* < 0.05, n = 172 oocytes; 40 µmol/L: 33.10 ± 0.82, *p* < 0.01, n = 163 oocytes; 60 µmol/L: 25.79 ± 1.93, *p* < 0.01, n = 135 oocytes) in comparison to the control group (65.20 ± 4.52, n = 132 oocytes). No significant difference was observed following the treatment with 2 μM TCS (55.14 ± 1.25, *p* > 0.05, n = 174 oocytes) (Figure 1B). Consequently, 20 µM of TCS was chosen for further investigations.

The developmental potential was then assessed by investigating the expansion of cumulus cells in relation to the effects of TCS. Figure 1C illustrates that most of the cumulus showed significant expansion in the control group, while many poorly expanded cumulus cells were detected following exposure to 20 µM TCS. To visually assess the expansion of the COCs, we also measured the levels of cumulus expansion quantitatively. The proportion of COCs categorized as Grade A showed a significant decline after exposure to 20 µM TCS (control: 47.06 ± 2.44, n = 266 COCs vs. TCS: 24.34 ± 1.89, n = 257 COCs, *p* < 0.01). In contrast, the proportion of COCs categorized as Grade C demonstrated a significant increase following exposure to 20 µM TCS (control: 21.55 ± 2.54, n = 266 COCs vs. TCS: 50.49 ± 3.22, n = 257 COCs, *p* < 0.01). Conversely, Grade B did not exhibit any significant difference between groups exposed to TCS and the control (control: 31.76 ± 2.17, n = 266 COCs vs. TCS: 25.18 ± 2.55, n = 257 COCs, *p* > 0.05) (Figure 1D). In general, the findings implied that TCS exposure could potentially hinder the maturation of porcine oocytes.

### 3.2. TCS Exposure Reduced the Antioxidant Capacity in Oocytes

**Figure 2:**
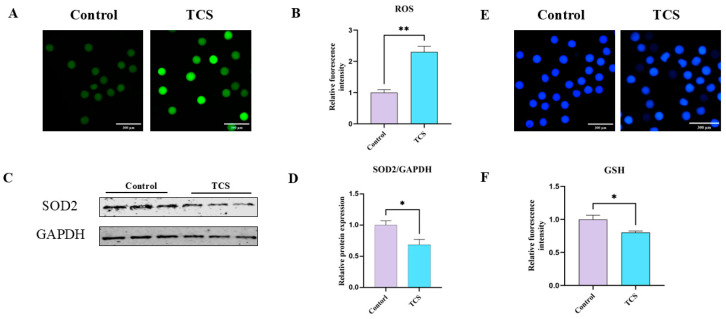
TCS exposure affects oxidative stress levels in oocytes. (**A**) Representative fluorescence images of ROS in control and TCS. Scale bar = 300 µm. (**B**) Quantitative analysis of ROS fluorescence intensity under control and TCS (** *p* < 0.01). (**C**) Protein expression of SOD2 and GAPDH was assessed in control and TCS. (**D**) Normalized SOD2/GAPDH expression ratios (* *p* < 0.05). (**E**) Representative GSH fluorescence images in control and TCS. Scale bar = 300 µm. (**F**) Relative GSH fluorescence intensity was quantified and compared between the control and TCS (* *p* < 0.05).

To examine the impact of TCS on oxidative stress in oocytes, we measured both the levels of ROS and the relative expression of proteins associated with antioxidants. The ROS signals in oocytes exposed to TCS showed significant differences when compared to the control group (Figure 2A). Furthermore, the quantitative assessment of ROS fluorescence intensity provided additional confirmation of these findings (control: 1.00 ± 0.10, n = 127 oocytes vs. TCS: 2.30 ± 0.18, n = 133 oocytes, *p* < 0.01) (Figure 2B). Additionally, the expression of antioxidant protein superoxide dismutase 2 (SOD2) was also determined by western blotting. The data revealed a significant decrease in SOD2 levels following TCS exposure (control: 1.00 ± 0.07 vs. TCS: 0.69 ± 0.08, *p* < 0.05, n = 360 oocytes) (Figure 2C,D). Excessive accumulation of ROS has the potential to deplete levels of the antioxidant glutathione (GSH), which is a crucial factor in the induction of oxidative stress. Therefore, we assessed GSH levels to understand this relationship better. The results, depicted in Figure 2E, clearly illustrated that the fluorescence signals corresponding to GSH in the oocytes were significantly reduced in the group exposed to TCS. Furthermore, a detailed analysis of the fluorescence intensity further corroborated this decrease, highlighting the impact of TCS exposure on GSH levels in oocytes (control: 1.00 ± 0.06, n = 150 oocytes vs. TCS: 0.80 ± 0.02, *p* < 0.05, n = 186 oocytes) (Figure 2F). These outcomes overall suggest that TCS induces oxidative stress in oocytes.

### 3.3. TCS Exposure Impairs Mitochondria Function in Oocytes

**Figure 3:**
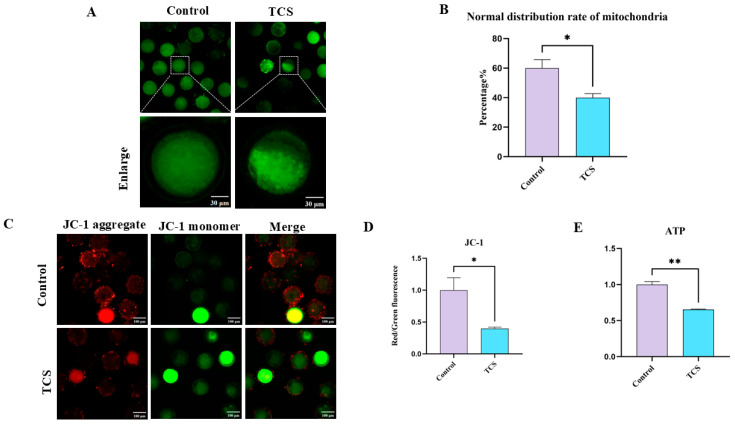
TCS affects the functioning of mitochondria in oocytes. (**A**) Representative micrographs show mitochondrial distribution from control and TCS. Scale bar = 30 μm. (**B**) The normal distribution rate of mitochondria was measured in control and TCS conditions (* *p* < 0.05). (**C**) Representative images illustrate ΔΨm in control and TCS conditions. Scale bar = 100 μm. (**D**) The red/green fluorescence ratio was quantified and compared between control and TCS conditions (* *p* < 0.05). (**E**) ATP levels were measured and compared between control and TCS conditions (** *p* < 0.01).

Initially, we evaluated mitochondrial distribution using Mito-Tracker staining. Mitochondria were uniformly distributed throughout the cytoplasm in control oocytes. Conversely, oocytes exposed to TCS displayed a reduction in mitochondrial signal in specific areas of the cytoplasm (Figure 3A). Analysis of the proportion of normal mitochondrial distribution further confirmed these findings (control: 60.02 ± 5.72, n = 80 oocytes vs. TCS: 39.90 ± 1.61, *p* < 0.05, n = 101 oocytes) (Figure 3B). Next, we assessed the mitochondrial membrane potential (ΔΨm) to examine mitochondrial functionality.

Figure 3C shows the representative images of mitochondrial ΔΨm. A significant reduction in ΔΨm was observed in TCS-exposed (0.40 ± 0.02, *p* < 0.05, n = 150 oocytes) relative to controls (1.00 ± 0.20, n = 107 oocytes) (Figure 3D). Mitochondrial impairment may lead to the inactivation of adenosine triphosphate (ATP); therefore, we measured ATP levels. As shown in Figure 3E, ATP concentrations were significantly lower in TCS-treated oocytes (0.65 ± 0.01, *p* < 0.01, n = 200 oocytes) compared to the control group (1.00 ± 0.04, n = 200 oocytes). Collectively, these findings indicated that TCS induces mitochondrial dysfunction in porcine oocytes.

### 3.4. TCS Exposure Affects the Endoplasmic Reticulum Function of Porcine Oocytes

**Figure 4:**
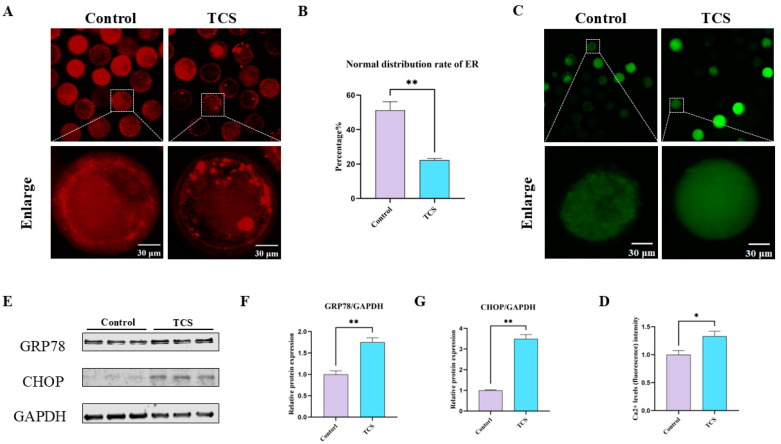
TCS exposure affects endoplasmic reticulum (ER) functions in oocytes. (**A**) Fluorescence micrographs depicting ER distribution in control and TCS-treated oocytes. Scale bar = 30 μm. (**B**) The normal distribution rate of ER was measured in control and TCS conditions (** *p* < 0.01). (**C**) Representative micrographs of Ca^2+^ in control and TCS. Scale bar = 30 µm. (**D**) Quantitative analysis revealed a significant increase in Ca^2+^ fluorescence intensity in TCS-treated oocytes (* *p* < 0.05). (**E**) Western blot analysis of GRP78, CHOP, and GAPDH protein expression in control and TCS. (**F**,**G**) Normalized expression ratios of GRP78/GAPDH and CHOP/GAPDH were significantly elevated in TCS-exposed oocytes (** *p* < 0.01).

The essential roles of the endoplasmic reticulum are associated with the formation of protein folding, secretion, and the regulation of cytoplasmic Ca^2+^ levels throughout oocyte maturation. This led us to explore the functions of the ER following exposure to TCS. Initially, we assessed the distribution of the ER utilizing ER-Tracker staining. ER fluorescence signals displayed a uniform distribution within the cytoplasm of control oocytes but significantly diminished following TCS exposure (Figure 4A). This reduction was corroborated by fluorescence intensity analysis (control: 51.29 ± 4.95, n = 81 oocytes; TCS: 22.26 ± 0.94, n = 90 oocytes, *p* < 0.01) (Figure 4B). Additionally, Ca^2+^ levels were measured using Fluo-4 AM staining. The results revealed significantly stronger Ca^2+^ signals in TCS-exposed oocytes compared to controls (Figure 4C). Intensity analysis confirmed a significant increase in fluorescence (control: 1.00 ± 0.07, n = 144 oocytes; TCS: 1.33 ± 0.09, n = 172 oocytes, * *p* < 0.05) (Figure 4D). Furthermore, protein expression levels of glucose-regulated protein 78 (GRP78) and C/EBP homologous protein (CHOP), markers of endoplasmic reticulum (ER) stress, were significantly elevated in TCS-exposed oocytes compared to controls (GRP78: 1.00 ± 0.08 vs. 1.75 ± 0.11, *p* < 0.01; CHOP: 1.00 ± 0.03 vs. 3.50 ± 0.21, *p* < 0.01; n = 360 oocytes) (Figure 4E–G). These results suggest that TCS exposure may lead to ER dysfunction in porcine oocytes.

### 3.5. TCS Exposure Induces Autophagic Functions in Porcine Oocytes

**Figure 5:**
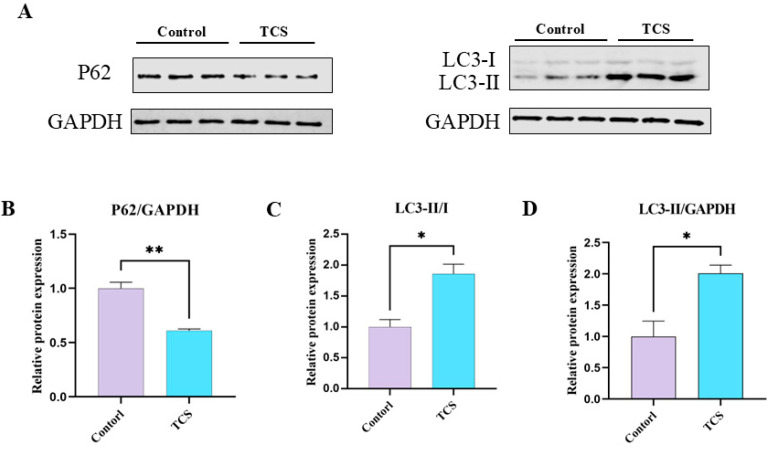
TCS exposure affects autophagy in oocytes. (**A**) Western blot analysis for the protein LC3-II/I, SQSTM1 (P62), and GAPDH expression in control and TCS. (**B**–**D**) The ratios of P62/GAPDH, LC3-II/I and LC3-II/GAPDH expression were normalized (* *p* < 0.05, ** *p* < 0.01).

Autophagy is a crucial mechanism responsible for the degradation of superfluous or malfunctioning cellular components. To explore the expression patterns of autophagic functions throughout porcine oocytes, we analyzed the levels of the autophagy marker proteins LC3 and P62 during the metaphase II (MII) stage of porcine oocytes using western blotting. As illustrated in Figure 5A, TCS exposure significantly increased the expression of LC3-II/GAPDH and LC3-II/I compared to the control groups, while P62 expression was significantly decreased. Quantitative analysis further confirmed these results (P62, control: 1.00 ± 0.06 vs. TCS: 0.61 ± 0.02, *p* < 0.01; LC3-II/I, control: 1.00 ± 0.12 vs. TCS: 1.86 ± 0.16, *p* < 0.05; LC3-II, control: 1.00 ± 0.24 vs. TCS: 2.01 ± 0.13, *p* < 0.05, n = 360 oocytes) (Figure 5B–D). Thus, these findings suggest that TCS exposure may impact autophagy functions in porcine oocytes.

### 3.6. TCS Promotes Early Apoptosis in Porcine Oocytes

**Figure 6:**
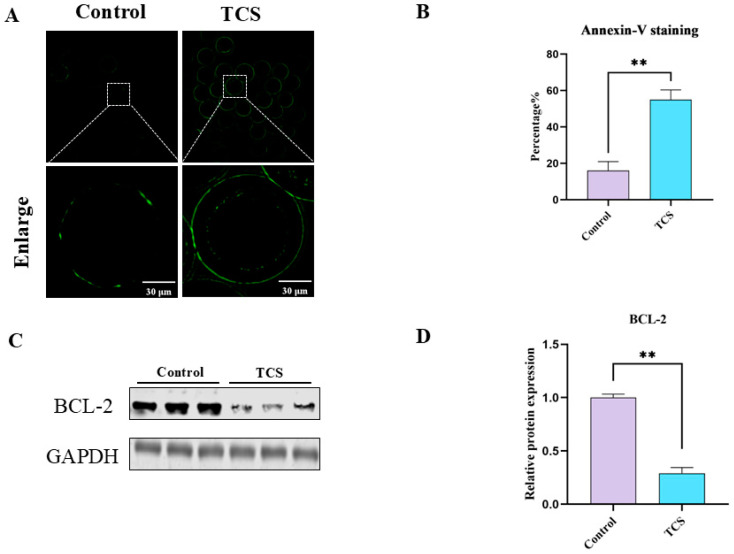
TCS exposure affects early apoptosis in oocytes. (**A**) Representative micrographs of early apoptosis were detected by Annexin-V in the control and TCS. Scale bar = 30 μm. (**B**) The ratios of early apoptosis were recorded in the control and TCS (** *p* < 0.01). (**C**) Western blot analysis for the protein BCL-2 and GAPDH expression in control and TCS. (**D**) The ratio of BCL-2 to GAPDH expression was normalized (** *p* < 0.01).

Green fluorescence on the oocyte membrane indicated early apoptosis, while faint signals were classified as negative (Figure 6A). TCS exposure significantly elevated the early apoptosis rate compared to the controls (control: 16.11 ± 4.87, n = 135 oocytes; TCS: 55.02 ± 5.31, n = 112 oocytes, *p* < 0.01) (Figure 6B). To investigate the mechanisms, BCL-2 expression was analyzed by western blotting. TCS-treated oocytes exhibited a significant reduction in BCL-2 levels (control: 1.00 ± 0.03 vs. TCS: 0.29 ± 0.05, *p* < 0.01, n = 360 oocytes) (Figure 6C,D). These results indicate that TCS may promote early apoptosis in porcine oocytes.

## 4. Discussion

The antibacterial agent triclosan (TCS) has garnered significant interest due to its adverse effects on animal health, particularly in relation to reproduction. Pigs exhibit metabolic and physiological similarities to humans, making them sensitive models for toxicity evaluation [24]. In this study, porcine oocytes were employed to investigate the effects of TCS on cytoplasmic functions during maturation. The results indicated that TCS exposure compromised oocyte quality through oxidative stress, impaired mitochondrial and endoplasmic reticulum function, dysregulated autophagy, and increased apoptosis.

The quality of oocyte maturation is a critical determinant of successful fertilization and subsequent embryonic development [25]. Key indicators of porcine oocyte maturation include cumulus cell expansion and polar body extrusion, which reflect cytoplasmic and nuclear maturation, respectively. Our study investigated the effects of TCS on these processes. In vitro experiments revealed that TCS exposure led to a concentration-dependent reduction in the first polar body extrusion, with 20 μmol/L, 40 μmol/L, and 60 μmol/L TCS significantly impairing oocyte maturation. Furthermore, cumulus cell expansion was markedly suppressed following treatment with 20 μmol/L TCS. Consistent with our findings, a previous study using porcine oocytes reported that 100 μmol/L TCS disrupted both meiotic progression and cumulus cell expansion, further highlighting its detrimental effects on oocyte maturation [26]. These observations prompted a deeper exploration of the molecular mechanisms underlying TCS-induced toxicity during porcine oocyte maturation.

Reactive oxygen can modify biological molecules, which can lead to abnormal development or even embryonic lethality [27]. Additionally, oxidative stress, which can be triggered by different stimuli based on their severity, has the potential to lead to either apoptosis or necrosis in both in vivo and in vitro environments [28]. Prior studies demonstrated that the generation of reactive oxygen species rose after treating primary mouse neurons with an exposure of 10 μmol/L TCS [29]. In vitro examinations of porcine granulosa cells demonstrated that treatment with 50 μmol/L TCS heightened oxidative stress while decreasing hormone synthesis [30]. Our findings revealed that 20 μmol/L TCS significantly elevated ROS levels, suggesting its role in inducing oxidative stress during the maturation of porcine oocytes. We have shown in the present study that TCS exposure reduces the levels of SOD2 and GSH in porcine oocytes. These findings are consistent with earlier reports, which observed a reduction in the expression and activity of antioxidant defense mechanisms under high or toxic TCS concentrations [31]. Nevertheless, mitochondrial dysfunction may serve as a potential factor associated with oxidative stress, prompting us to further investigate the mitochondrial functions of TCS throughout the maturation of porcine oocytes.

Proper organelle distribution and function are essential for successful oocyte maturation [32]. Previous research has indicated that abnormalities in mitochondrial localization may lead to mitochondrial dysfunction [33]. Several studies have reported that TCS treatment alters mitochondrial morphology [34]. Our results showed a significant decrease in the number of evenly distributed mitochondria following TCS exposure. In the mouse microglial cell line (BV-2), treatment with 20 μM TCS led to elevated ROS levels, reduced ΔΨm, and subsequent mitochondrial dysfunction [35]. Furthermore, TCS impaired mitochondrial function by disrupting MMP in Zebrafish and, ultimately, ATP synthesis [31]. As a result, we evaluated ΔΨm and ATP following exposure to TCS, with findings revealing that TCS exposure diminished levels of both ΔΨm and ATP. These results imply that TCS exposure triggers mitochondrial dysfunction, ultimately resulting in oxidative stress within porcine oocytes.

Considering the essential role of the ER in the meiotic maturation of oocytes, particularly through its influence on the regulation of cytoplasmic Ca^2+^ levels and the biogenesis of protein synthesis, folding, and maturation, any alterations in the distribution of the ER may indicate interference with these vital functions [36]. Disruptions in ER distribution may impair these critical functions. Previous studies have shown that TCS exposure in stem cells from human exfoliated deciduous teeth (SHED) caused mitochondrial dysfunction and endoplasmic reticulum stress [37]. To investigate ER function in TCS-exposed oocytes, we first assessed ER distribution. Our findings revealed abnormal ER distribution in the cytoplasm of TCS-treated oocytes. As the ER is a primary Ca^2+^ storage site, and Ca^2+^ signaling is essential for meiotic communication [38], we measured cytoplasmic Ca^2+^ levels. TCS exposure significantly increased intracellular Ca^2+^ signals, indicating ER dysfunction. Furthermore, similar to the effects of zearalenone, which activates CHOP and GRP78 expression to induce ER stress in porcine oocytes [39], TCS exposure increased GRP78 and CHOP levels in our study. Both proteins are key mediators of the unfolded protein response (UPR) and ER stress pathways [40]. Additionally, TCS has been shown to disrupt ER signaling in mast cells by modulating calcium homeostasis. These findings suggest that TCS disrupts ER homeostasis and function, potentially compromising porcine oocyte maturation.

The process of autophagy in oocytes is crucial to the mechanisms underlying significant follicular atresia [41]. Prior research has demonstrated that autophagy occurs during the in vitro maturation of porcine oocytes, varying over time within cultures [42,43]. Consequently, we investigated the effects of TCS on autophagic activity during porcine oocyte maturation. Our results revealed that TCS exposure increased autophagic activity, as evidenced by LC3-II accumulation and P62 degradation in porcine oocytes. A similar finding indicated that TCS triggers autophagy through the AMPK/LC3/p62 signaling pathway, resulting in increased LC3 protein expression and decreased P62 protein expression. This suggests that TCS promotes autophagy and cell death [44]. Overall, our findings indicate that exposure to TCS stimulates autophagy, which subsequently adversely affects the maturation quality of porcine oocytes.

Recent research indicates that both autophagy and apoptosis can be simultaneously triggered by various stress factors, sharing several regulatory molecules and facilitating their interchange [45,46]. In human oral squamous cell carcinoma cells (SCC-15), ROS-dependent apoptosis was triggered by 10 μmol/L TCS through the activation of caspase-3 [47]. Early apoptotic events, assessed using the Annexin V-FITC assay, revealed that exposure to TCS led to early apoptotic changes in porcine oocytes. The ratio of Bax to Bcl-2 activates intrinsic apoptotic pathways [48]. It has been reported that the antiapoptotic protein Bcl-2 exhibited a downward trend in response to 10 μmol/L TCS, promoting apoptosis in HT-22 cells [49]. Consistent with these findings, our results indicated a significant decrease in Bcl-2 protein expression in TCS-exposed oocytes. Given that connections between apoptosis and autophagy markers are present in all estrous cycle phases, including dying oocytes [50], early apoptosis induced by TCS exposure may influence porcine oocyte maturation through autophagy.

Furthermore, the transgenerational implications of TCS exposure on embryonic development and offspring health warrant rigorous investigation. Emerging evidence indicates that gestational and lactational administration of TCS induces multigenerational reproductive impairments, including reduced fertility in F1 male offspring and developmental anomalies in F2 offspring, resulting in outcomes such as decreased fetal weight and reduced crown-rump length [51]. Chronic exposure to environmentally relevant concentrations of TCS (specifically 2–200 μg/L) has been shown to exacerbate reproductive toxicity in aquatic models. This exposure significantly depletes germ cell reserves and compromises offspring survival rates [13]. Notably, early life TCS exposure disrupts thyroid homeostasis by suppressing the synthesis of triiodothyronine (T3) and thyroxine (T4). This disruption is associated with delayed embryonic hatching and pathological thyroid follicular remodeling, including hyperplasia and angiogenesis [52]. Collectively, these findings underscore TCS as a potent endocrine disruptor capable of eliciting transgenerational defects through interconnected mechanisms.

## 5. Conclusions

Our findings demonstrate that TCS exposure adversely affects porcine oocyte maturation through multiple pathways. The observed reduction in maturation rates and cumulus expansion suggests that TCS compromises oocyte developmental competence. This is likely mediated by mitochondrial dysfunction, as evidenced by ROS accumulation, decreased antioxidant enzyme expression, and loss of ΔΨm. Furthermore, TCS disrupted ER function and Ca^2^⁺ homeostasis, leading to ER stress. The induction of autophagy and apoptosis further underscores the cytotoxic effects of TCS on oocytes. These results align with previous studies showing that environmental toxins can impair reproductive health by targeting cytoplasmic functions. Further studies are needed to investigate the long-term consequences of TCS exposure on embryo development and offspring health.

## Data Availability

The data presented in this study are available on request from the corresponding author.
